# Biodegradable Carbonate Apatite Nanoparticle as a Delivery System to Promote Afatinib Delivery for Non-Small Cell Lung Cancer Treatment

**DOI:** 10.3390/pharmaceutics14061230

**Published:** 2022-06-10

**Authors:** Nian N. N. Maarof, Emilia Abdulmalek, Sharida Fakurazi, Mohd Basyaruddin Abdul Rahman

**Affiliations:** 1 Integrated Chemical BioPhysics Research, Department of Chemistry, Faculty of Science, Universiti Putra Malaysia, Serdang 43400, Selangor, Malaysia; gs53758@student.upm.edu.my (N.N.N.M.); emilia@upm.edu.my (E.A.); 2 Department of Chemistry, College of Education, University of Sulaimani, Sulaimani 46001, Iraq; 3 Laboratory of Vaccines and Immunotherapeutics, Institute of Bioscience, Universiti Putra Malaysia UPM, Serdang 43400, Selangor, Malaysia; sharida@upm.edu.my; 4 Department of Human Anatomy, Faculty of Medicine and Health Sciences, Universiti Putra Malaysia UPM, Serdang 43400, Selangor, Malaysia; 5 UPM-MAKNA Cancer Laboratory, Institute of Bioscience, Universiti Putra Malaysia, Serdang 43400, Selangor, Malaysia

**Keywords:** nanomedicine, carbonate apatite nanoparticle (nCA), afatinib, drug delivery, lung cancer

## Abstract

Nanomedicine-based drug-delivery systems have significant interest in cancer treatment, such as improving the stabilities and biocompatibilities, precise targeting, and reducing toxicities for non-cancerous cells. Herein, this study presents the synthesis and characterisation of carbonate apatite nanoparticles (nCA) and encapsulated afatinib (AFA) as promising drug delivery candidates for lung cancer treatment. nCA/AFA was synthesised and physicochemically characterised, then the encapsulation capacity, drug loading, and cumulative drug release profile were evaluated. Powder X-ray diffraction (PXRD) confirmed that the synthesised nCA is apatite. Fourier-transform infrared spectroscopy (FTIR) results confirmed the drug loading into the nanoparticles. High-resolution transmission electron microscopy (HR-TEM) determined the morphology of nCA and nCA/AFA and the diameters of 47.36 ± 3.16 and 42.97 ± 2.78 nm, respectively, without an unaltered nCA phase. Encapsulation efficiency (%) and drug loading (%) were 55.08% ± 1.68% and 8.19% ± 0.52%. Brunauer–Emmett–Teller (BET) and dynamic light-scattering (DLS) results revealed that the synthesised nCA is mesoporous, with a surface area of 55.53 m^2^/g, and is negatively charged. Atomic force microscopy (AFM) showed increasing roughness of nCA/AFA compared to nCA. The drug release from the nano-formulation nCA/AFA demonstrated slow and sustained release compared to the pure drug. Accordingly, nCA/AFA represents a promising drug delivery system for NSCLC treatment.

## 1. Introduction

Lung cancer is the second most diagnosed cancer worldwide. In 2020, according to the Global Cancer Observatory (GCO), lung cancer incidence was 2.2 million (11.4%), and mortality was 1.8 million (18%) [1]. Histologically, lung cancer is divided into two main types, with the majority (≈85%) having non-small cell lung cancer (NSCLC), and 15% with small cell lung cancer (SCLC). Pathologically, NSCLC is classified into three major classes: adenocarcinoma, large cell carcinoma, and squamous cell carcinoma [2]. These subtypes of NSCLC are strongly correlated with smoking [3]. NSCLC is characterised by different types of mutations, including epidermal growth factor receptor (EGFR), Kirsten rat sarcoma viral oncogene homolog gene (KRAS), and rare anaplastic lymphoma kinase gene (ALK) [4]. EGFR has been detected in 30–40% of NSCLC, defined as a transmembrane glycoprotein with cytoplasmic kinase activity involved in cancer cell proliferation, angiogenesis, and metastasis [5]. Conventionally, the most common strategies for lung cancer treatment involve surgery, radiotherapy, and chemotherapy. Chemotherapy is the major lung cancer treatment method, specifically for advanced stages in which the drugs are delivered orally or intravenously. However, the limitation of the chemotherapeutic drugs is related to their systemic side effects, lack of selectivity, non-localised delivery of drugs to the target site, and development of drug resistance towards cancerous cells [6,7]. Nanomedicine is a nanotechnology application for medical purposes, and it has the potential to improve cancer treatment outcomes [7]. Of specific interest is the utility of nanoparticles in drug-delivery applications [8].

Carbonate apatite is an inorganic pH-sensitive nanocarrier with a chemical formula of (Ca)_10_(PO_4_)_6-x_(CO_3_)_x_(OH)_2_. It has a heterogeneous surface charge distribution, biodegradability, and the ability to prevent crystal growth for the production of nanoscale particles [9]. nCAs have rich Ca^2+^, HCO^3−^, and PO_4_^3−^ domains that provide various possibilities for both negatively and positively charged drugs to bind via ionic interactions [10]. nCA/AFA nanocomposites can enter lung cancer cells via endocytosis, discharging the loading drug after nanoparticle degradation in endosomal acidic environments. In addition, various chemotherapeutics [10] and genetics [11] have been successfully loaded into nCA. 

Interestingly, afatinib (AFA) is a second-generation tyrosine kinase inhibitor (TKI) with high selectivity in inhibiting the ErbB family through covalent and irreversible binding to epidermal growth factor receptors (EGFR), making afatinib a potent treatment [12], which could stand by itself as a safe and effective first- and second-line monotherapy for survival improvement of NSCLC patients [13]. Afatinib inhibition mechanisms indicate a reduction in the autophosphorylation of EGFR by blocking their downstream signalling pathways. Afatinib covalently links to its ATP-binding site via the acrylamide site of the afatinib and residues of cysteine (Cys 797, Cys 803, and Cys805) of epidermal growth factor, human epidermal growth factor 2, and human epidermal growth factor 4, respectively, subsequently inhibiting the autophosphorylation of the tyrosine kinase and EGFR downregulating signalling pathway [14]. Some of the most common EGFR mutations are exon 19 deletions (Del19) and L858R point mutations (L858R) [15]. Tumours with EGFR activation mutations might entirely depend on the activity of EGFR to promote the signalling pathway. In these cases, blockage of EGFR with afatinib inhibits the proliferation and induces apoptosis [16]. However, afatinib is a hydrophobic drug that has low bioavailability, leading to severe side effects and high distribution throughout the body [17]. In addition, a strong irreversible attraction between afatinib and EGFR may be revealed in non-cancerous cells (normal cells), increasing the adverse side effects of afatinib, such as gastrointestinal (GI) symptoms, pulmonary symptoms, and others [18]. Rash, breathing trouble, grade 3–4 diarrhoea, or acne are some examples of some serious afatinib side effects which may occur [19]. Additionally, afatinib is an orally administered drug, and after several doses, the GI symptoms can worsen [20]. Hence, this study develops an appropriate system for delivering afatinib to non-small cell lung cancer treatment that reduces its side effects and promotes anti-cancer effects.

**Figure 1:**
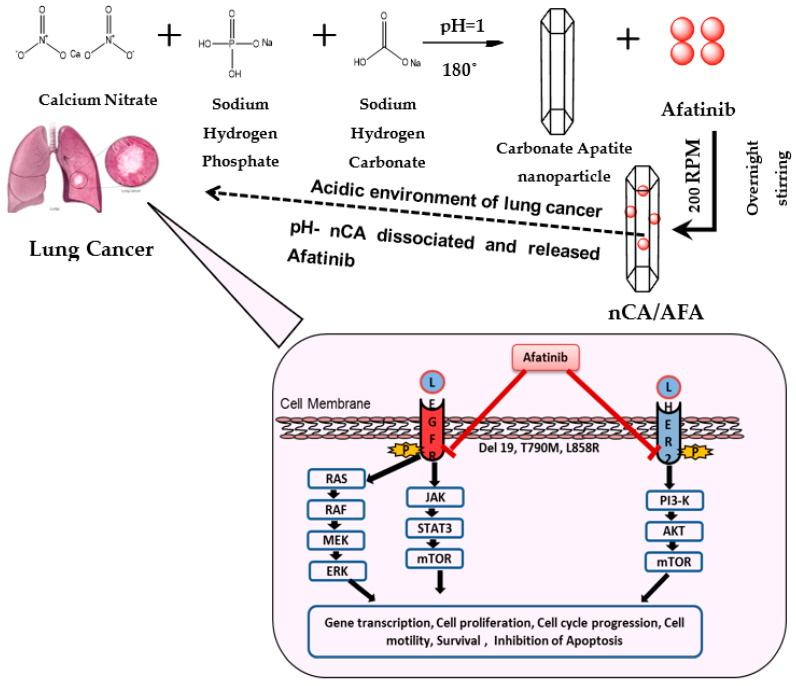
Schematic illustration of nCA/AFA nanocomposite synthesis and mechanism of action for targeted cancer cells. nCA/AFA nanocomposites are relatively stable at physiological pH, but they are dissociated in the acidic pH of cancer cells and the release of the afatinib drug. Afatinib is covalently bound to the ATP-binding site of the tyrosine kinase of EGFR, irreversibly inhibiting its autophosphorylation and leading to downregulation of the signalling pathways. As a result, it induces apoptosis and reduces cancer cell survival and proliferation.

This study reported synthesising nanocomposites (nCA/AFA), as shown in Figure 1, as a cheap, biodegradable, biocompatible, and not toxic nanocarrier-encapsulated afatinib drug for targeted treatment of NSCLC. The aim of the current work is to explore afatinib drug encapsulation into nCA, which has not been reported previously.

There have been many previous attempts to encapsulate afatinib into other nanocarriers, such as Elbatanony et al., who encapsulated afatinib into PLGA nanoparticles to treat NSCLC. The result demonstrated higher cytotoxicity enhancement towards the NSCLC cell line in vitro and increased cellular uptake. However, nCA/AFA showed an improvement in cumulative release of 40.1% for nCA/AFA compared to 56.8% in 48 h for PLGA/AFA in PBS medium at pH 7.4 [21]. Additionally, Hong et al. used lipid polymeric nanoparticles to encapsulate afatinib and microRNA in the treatment of colorectal cancer. The final formulation has significantly inhibited the migration and resistance of colorectal cancer cell lines of induced apoptosis by blocking the metastasis pathway (HER-tyrosine kinase pathway) [22].

The physicochemical characterisations of the nanoparticles and nanocomposites are determined to evaluate their biological effect and long-term stability [23]. The successful formation and the excellent results provide a powerful drug-delivery system for lung cancer treatment.

## 2. Materials and Methods

### 2.1. Materials

Calcium nitrate tetrahydrate (Ca(NO_3_)_2_·4H_2_O) (min. 99%, analytical reagent, Sigma-Aldrich), sodium dihydrogen phosphate monohydrate (NaH_2_PO_4_.H_2_O) (min. 99%, analytical reagent, Merck, Darmstadt, Germany), sodium hydrogen carbonate (NaHCO_3_, 99%, Merck), afatinib (purity 99.9% Biosyntech, ‘s-Hertogenbosch, The Netherlands), phosphate buffer saline (Sigma-Aldrich), ethanol (purity 99.8%, Sigma-Aldrich, St. Louis, MO, USA), simulated lung fluid (LCTech, Obertaufkirchen, Germany), and bovine albumin serum (purity > 96%, Merck) were used.

### 2.2. Preparation of nCA Nanocarriers and nCA/AFA Nanocomposites

The synthesis of nCA was comprised hydrothermally via adding 0.42 g of sodium bicarbonate (NaHCO_3_) into 50 mL (0.3 M) of sodium hydrogen phosphate (NaH_2_PO_4_.H_2_O). The phosphate to carbonate molar ratio is 3:1. The mixed solution of NaH_2_PO_4_.H_2_O and NaHCO_3_ was added dropwise into a solution of 50 mL (0.5 M) of calcium nitrate tetrahydrate (Ca(NO_3_)_4_.4H_2_O) under intense stirring. The pH of the new mixture was maintained by a pH meter at 12 by using 1 M sodium hydroxide (NaOH) solution. The mixed solution was stirred at 60 °C for 2 h, then transferred to a Teflon stainless-steel autoclave and tightly sealed for 12 h in the electric oven at 180 °C for the hydrothermal reaction. When the reaction was completed, the precipitate was collected via centrifugation at 10,000 rpm for 20 min, washed with deionised water (3 × 10 mL), and dried at 100 °C for 10 h [24]. The resultant powder was characterised prior to afatinib drug loading.

nCA/AFA nanocomposites with various afatinib masses (150, 500, 750, and 1000 µg) were prepared, and 5 mg of nCA was suspended into 5 mL of ethanol. Then, the solution of afatinib was added dropwise. The mixture was stirred at 200 rpm overnight in the dark at room temperature. nCA/AFA nanocomposites were obtained after centrifugation for 10 min at 1000 rpm, washed with ethanol (3 × 10 mL), and dried.

### 2.3. Characterisation of nCA and nCA/AFA

Physicochemical characterisation of nCA and nCA/AFA was conducted by atomic force microscope (AFM), zeta potential and hydrodynamic diameter detection, Fourier-transform infrared spectroscopy (FTIR), field emission scanning electron microscopy (FESEM), high-resolution transmission electron microscopy (HR-TEM), Brunauer–Emmett–Teller (BET) analysis, and powder X-ray diffraction (PXRD).

For PXRD, the samples were scanned with a diffraction angle from 3° to 50° by using the Cu X-ray tube at radiation λ = 1.540598 Å of the Shimadzu XRD-6000 powder diffractometer. The PXRD patterns were obtained using the Shimadzu XRD-6000 powder diffractometer with Cu K (=1.540562 Å) as the X-ray source. The samples were scanned at the step size of (2 Th.) 0.033 and scan step time (s) of 19.685, with diffraction angles ranging from 3° to 50°, a voltage of 40 kV, and a current of 40 mA. X‘Pert HighScore Plus software was used for the analysis. FTIR spectra were performed using a Nicolet 6700 (ATR) attenuated total reflection spectrophotometer between 500 and 3500 cm^−1^.

The HR-TEM measurements were determined using JEOL 2010F HR-TEM. Sample preparations were performed by dispersing certain amounts into ethanol and placing them onto a carbon-coated copper TEM grid. Then, a heat lamp was used to dry and measure. For FESEM, samples were prepared on a 12 mm-diameter aluminium holder, then vacuum-coated with a platinum layer and examined. Image J software was used to analyse all images.

Atomic force microscope (Bruker Dimension EDGE AFM) was used for surface imaging in tapping mode at a scanning rate of 0.9 Hz. The samples were prepared by suspending them in ethanol and sonicating for 10 min, and then they were transferred onto a glass slide followed by air-drying at room temperature. NanoScope analysis 1.7 was used for the analysis of images.

For hydrodynamic diameter, polydispersity index, and zeta potential, 0.2 mg of each sample was sonicated for about 15 min after being dispersed in 20 mL of deionised water. Then, the sample was transferred into disposable cuvettes for measurements using a light-scattering (Zetasizer Nano ZS Malvern dynamic DLS) instrument. Finally, the surface area measurement was determined by the N_2_ adsorption and desorption experiment that was carried out by degassing all samples for 8 h at 120 °C by using Micromeritics Tristar II instruments.

### 2.4. Yield, Encapsulation Efficiency, and Drug Loading

The obtained yield of nCA/AFA was calculated using Equation (1) [25]:

(1) $$Yeild=\frac{\mathrm{Mass}\;\mathrm{of}\;\mathrm{nCA}/\mathrm{AFA}\;\left(\mathrm{mg}\right)}{[(\mathrm{Mass}\;\mathrm{of}\;\mathrm{nCA}\;\left(\mathrm{mg}\right)+\left(\mathrm{Mass}\;\mathrm{of}\;\mathrm{AFA}\;\left(\mathrm{mg}\right)\right]}\times 100$$

A UV-Vis spectrophotometer was used to collect and measure the supernatant concentration of nCA/AFA nanocomposites at maximum absorbance measurements (λ_abs_ = 337 nm) to calculate the amount of un-entrapped AFA. As the initial amounts of AFA and nCA were known, the encapsulation efficiency (EE%) and drug loading (DL%) were calculated as an average of three measurements for each experiment using Equations (2) and (3) [26]:

(2) $$\mathrm{Encasulation}\;\mathrm{Efficiency}\;\left(\mathrm{EE}\right)\%=\frac{\mathrm{Mass}\;\mathrm{of}\;\mathrm{encapsulated}\;\mathrm{AFA}\;\left(\mathrm{mg}\right)}{\mathrm{The}\;\mathrm{initial}\;\mathrm{mass}\;\mathrm{of}\;\mathrm{AFA}\;\left(\mathrm{mg}\right)}\times 100$$

(3) $$\mathrm{Drug}\;\mathrm{Loading}\;\left(\mathrm{DL}\right)\%=\frac{\mathrm{Mass}\;\mathrm{of}\;\mathrm{encapsulated}\;\mathrm{AFA}\;\left(\mathrm{mg}\right)}{\mathrm{Mass}\;\mathrm{of}\;\mathrm{nanocarriers}\;\left(\mathrm{mg}\right)}\times 100$$

### 2.5. Release Study

For in vitro drug release, 5 mg of nCA/AFA and 400 µg of free AFA were placed separately into 1 mL of ethanol into Fisher brand dialysis tubing (12,000–14,000 MWCO) and suspended in 25 mL of PBS (pH 7.4 and 5.5) at 37 °C, with mild agitation at 100 rpm. Then, 1 mL of the samples was collected and substituted with fresh buffer at the scheduled time interval. The released media were determined using a UV-Vis spectrometer for absorbance measurements (λ_abs_ = 337 nm).

### 2.6. Dissolution Analysis of nCA in Biological Media

The dissolution of nCAs was measured in simulated plasma solution (SPS) and simulated lung fluid (SLF) in physiological (pH 7.4) and tumour acidic (pH 5.5) conditions. The SPS was made by dissolving 10 g of bovine albumin serum in 200 mL of phosphate-buffered saline. Then, 50 mg of nCA was dispensed in 30 mL of the above-mentioned media at pH 7.4 and 5.5, as mentioned by Chemmalar et al. [27]. The nCAs were stirred in a thermostatic shaker at 100 rpm and 37 °C. At a certain time interval, 10 mL of supernatants was collected and centrifuged at 6000 rpm for 15 min, and the same volume of media was added to the tubes. Analyses for total calcium content in each media were performed using inductively coupled plasma—optical emission spectrometry (Perkin Elmer, Optima 2000DV, ICP-OES).

### 2.7. Statistical Analysis

The collected data are presented as the mean and standard deviation (SD). The XRD peaks’ full width at half-maximum (FWHM) was analysed using the fitting Gauss and Lorentz function. All statistics were calculated by using OriginPro 9.0 software (OriginLab, Northampton, MA, USA). ANOVA was used to determine the statistical difference of parameters using Tukey’s and Bonferroni’s model for a *p*-value less than 0.5 to be considered significant.

## 3. Results and Discussion

### 3.1. Characterisation of nCA and nCA/AFA

#### 3.1.1. XRD Analysis

**Figure 2:**
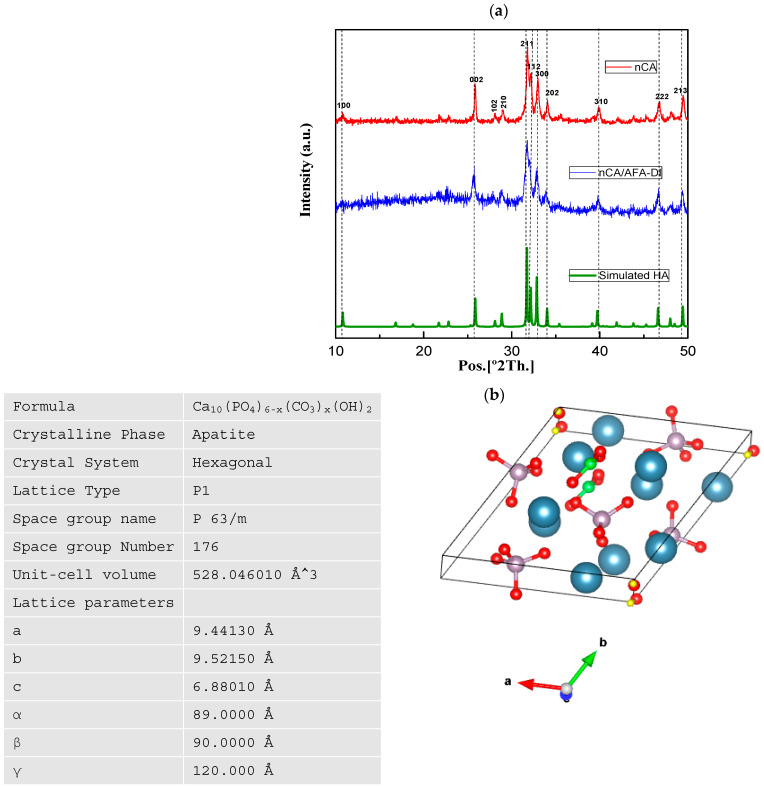
PXRD spectra of nCA and nCA/AFA. (**a**) PXRD patterns of nCA (red), nCA/AFA (blue), and simulated HA (green). (**b**) Hexagonal crystalline morphology of the synthesised nCA, with calcium (blue), phosphorous (purple), carbon (green), oxygen (red), and hydrogen (yellow), using the Vesta program and its lattice parameter.

The XRD technique helps to identify the nanoparticles’ crystallinity, texture, and phase analysis [28]. The XRD patterns of nCA and nCA/AFA are presented in Figure 2, and the theoretical diffraction pattern is simulated from a single crystal of hydroxyapatite (HA). 

For the hydrothermally synthesised nCA, all peaks are assigned to the HA phase without any extra reflections, which shows that the addition of the carbonate group has no significant change compared to the HA structure. However, diffraction peaks with line broadening and high intensities in planes of 002, 211, 112, and 300 confirm the nano-size and change crystallinity of the nCA due to the addition of the CO_3_^2−^ group. The hkl diffraction plane suggests hexagonal crystalline morphology of nCA, and it is matched with JCPDS Card No. 09-0432. Further, the peaks of nCA/AFA can be noticed without any impurity peaks, which confirms the product’s purity and accuracy.

The crystal structure and the lattice parameters were obtained from the synthesised nCA (Figure 2b). The average crystalline sizes of the nCAs were calculated by using Scherrer’s equation:

(4) $$Dhkl=\frac{kh}{B_{1/2}{\cos\theta }_{hkl}}$$

where D is the size of the crystallite, *k* with the value of 0.94 is the shape factor, and λ is equivalent to the wavelength of CuKα (0.15418 nm). *B*_1/2_ corresponds to the FWHM of the plane, and θ*hkl* is the Bragg angle (°). The highest intensity diffraction peak at 211 was chosen to determine the crystallite size. According to Scherrer’s equation, the average crystallite sizes of nCA and nCA/AFA were 46.95 and 42.46 nm, respectively.

#### 3.1.2. FTIR Analysis

**Figure 3:**
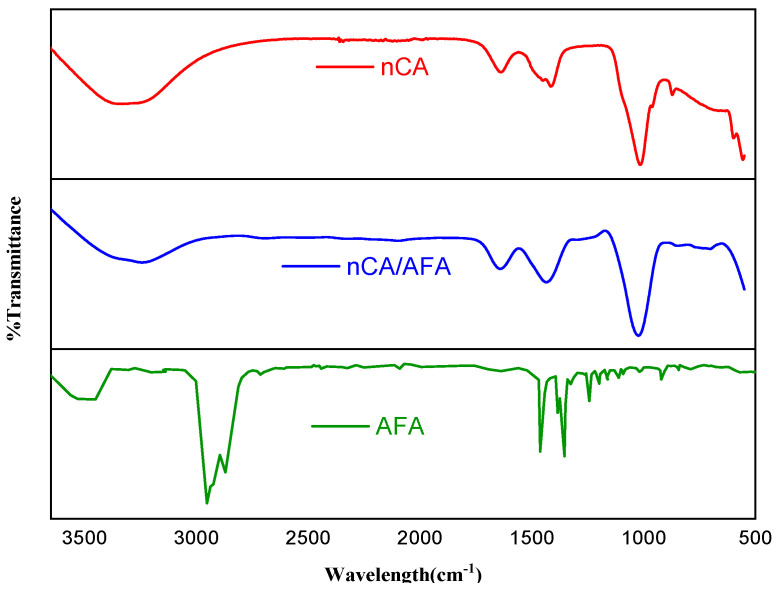
FTIR spectra for nCA (red), nCA/AFA (blue), and AFA (green). The transmittance changes due to afatinib loading into nCA are mentioned in the green box.

For drug-delivery research, FTIR studies the purity, surface chemical analysis, and degree of composites’ conversion [29,30]. In this study, FTIR was used to identify the characteristic nCA function groups, namely, CO_3_^2−^, PO_4_^3−^, and OH^−^. The spectra of nCA, nCA/AFA, and pur AFA are shown in Figure 3, and the full spectra ranged from 500 to 3500 cm^−1^. The bending mode of the PO_4_^3−^ group can be noticed at ~565 and 808 cm^−1^. The υ_1_ symmetric stretching of the PO_4_^3−^ group can be observed at the shoulder at ~965 cm^−1^ [27,28]. The main band of the υ_3_ asymmetric stretching mode of the PO_4_^3−^ group can be characterised between 1000 and 1100 cm^−1^. The presence of the CO_3_^2−^ group is confirmed by the υ_1_ vibrational signature around ~1415 and 1473 cm^−1^ and the υ_2_ vibration mode of the CO_3_^2−^ group at 873 cm^−1^. The broad absorption band at ~3752 cm^−1^ is related to the stretching vibration of the OH^−^ group. These findings are similar to FTIR data reported for carbonate apatite nanoparticles, as mentioned in previous works [31,32]. The band shoulders at ~875 and 1419 cm^−1^ for carbonate were modified after drug loading. The OH^−^ band became broader, and a new low-intensity peak at 2810 cm^−1^ appeared due to the effect of afatinib on nCA.

The AFA spectrum characteristic peaks appeared at 2833 and 2970 cm^−1^, representing C-H stretching of the methyl group (CH_3_). Additionally, the C=O stretching of the amide group can be demonstrated at 1647 cm^−1^, and C-N stretching of aromatic amine can be seen at 1355 cm^−1^. For the nCA/AFA, there were some decreases and variations in transmittance, as highlighted by the green in Figure 3, which can be explained by the presence of more bonds in the case of the transmittance variation between nCA and nCA/AFA. For nCA/AFA, there were no peaks detected in the same region compared to nCA alone, which may confirm the AFA loading into the nCA, rather than outside. 

#### 3.1.3. HR-TEM Analysis

**Figure 4:**
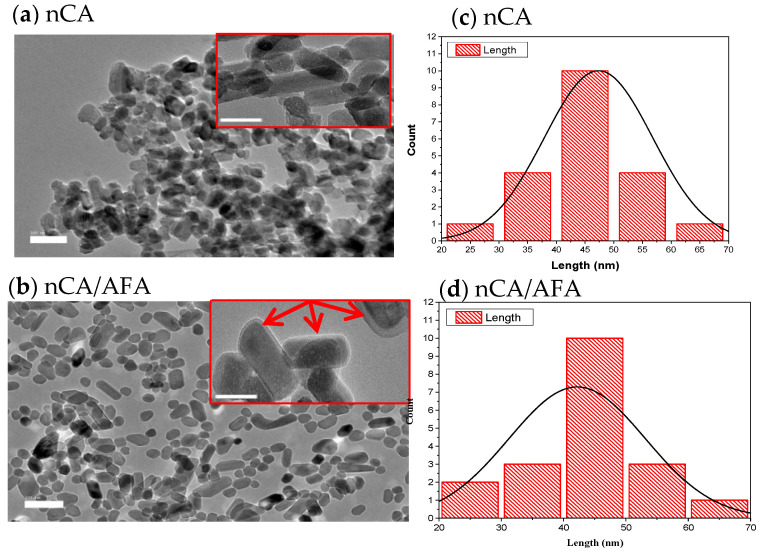
HR-TEM images. (**a**,**b**) HR-TEM micrographs of nCA and nCA/AFA with a scale bar of 100 nm. Each image inset has a scale bar of 20 nm, and the red arrow confirms the encapsulation of the drug into the nCA. (**c**,**d**) Histogram of nCa and nCA/AFA size as measured by HR-TEM (Gaussian distribution size).

The HR-TEM analysis was used to determine the size and shape of nCA and nCA/AFA, as shown in Figure 4. The observed morphology of the nCAs shows a rod-like crystalline, uniform porous surface structure with an average diameter of 47.36 ± 3.16 nm. Adding afatinib to nCA decreases the particle size to the average diameter of 42.97 ± 2.78 nm, which may confirm AFA loading into nCA as it will reduce the aggregation of the nCA (Figure 4a,b). This can be attributed to the drug, which may act as a surfactant and prevent aggregation of nCA [33,34,35]. Furthermore, the measurement size obtained for nCA and nCA/AFA agreed with the Gaussian distribution size obtained for nCA and nCA/AFA, ≈47 and 42.61 nm, respectively (Figure 4c,d).

#### 3.1.4. FESEM and Morphology Analysis

**Figure 5:**
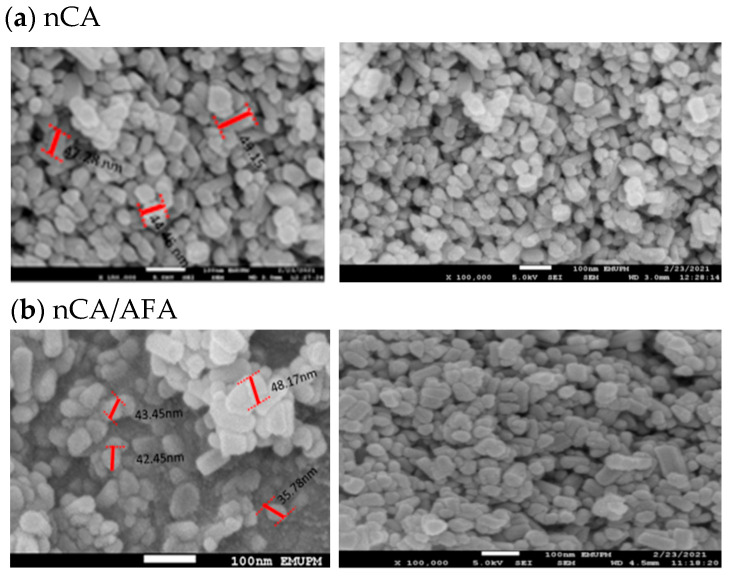
FESEM representative images of (**a**) nCa and (**b**) nCA/AFA, with a scale bar of 100 nm.

The surface morphological characterisation of nCA and nCA/AFA from FESEM graphs is shown in Figure 5. The nCA demonstrated agglomerated, nearly rod-like shaped nanoparticles, and this fine agglomeration is due to the van der Waals force attraction between nCAs. 

The average length, according to FESEM, for the nCA was 49.98 ± 5.71 nm, and 22.65 ± 1.94 nm in width. Unchanged morphology for nCA/AFA could be noticed but with reduced length and width as a function of afatinib drug encapsulation, where the length was 42.47 ± 4.72 nm, and the width was 21.58 ± 1.77 nm.

#### 3.1.5. AFM and Topography Analysis

**Figure 6:**
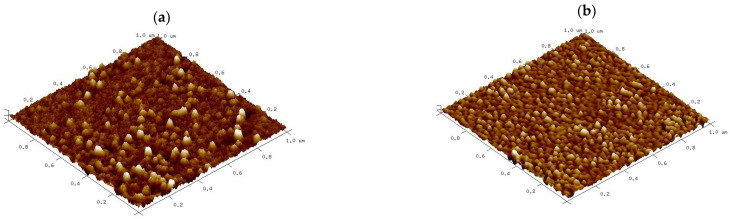
Three-dimensional reconstructions of (**a**) nCA and (**b**) nCA/AFA based on 1 × 1 μm atomic force microscopy (AFM) scans.

The surface architecture and the topography of nCA and nCA/AFA were analysed via AFM. Figure 6 shows three-dimensional images scanned using tapping mode over a 1 × 1 µm^2^ surface area. The nCA image in Figure 6a demonstrates nearly spherical nanoparticles with a diameter of 31.63 nm. At the same time, nCA/AFA showed more fine spherical granular surfaces distributed highly uniformly within a diameter of 27.48 nm, as shown in Figure 6b. The surface architecture in vertical dimensions was determined quantitatively by the roughness. Several roughness parameters were considered, as shown in Table 1, such as the average surface roughness (Ra), the maximum surface roughness (Rmax), and the root mean square roughness (Rq). Other parameters, such as surface area differences (Rsa), Kurtosis, and Skewness, were used for horizontal dimension description [36]. The average roughness (Ra) value of nCA was nearly the same as the value of nCA/AFA, indicating that the afatinib drug was encapsulated inside the nCA rather than on the surface. The Rmax is the maximum difference between the highest peak and lowest valleys, and it was larger, with 7.8 nm in nCA compared to 7.2 nm in nCA/AFA.

Skewness is the measure of profile symmetry around the mean lines, while Kurtosis measures the sharpness of the roughness profile around the mean. For nCA, the Skewness was 1.90, while it was smaller in nCA/AFA, only 0.97, which indicates a symmetrical Skewness. The typical height distribution should be 3 to be considered normal for Kurtosis [37]. The value for nCA was 3.89, but nCA/AFA had a distribution with a relatively normal value of 3.05. Indeed, the morphology and roughness are essential as the rougher surface character of nanoparticles is generally associated with a higher potential for inhibiting certain cancer cell types [38]. As previously shown in the study of Xue et al., by increasing polydopamine nanoparticle surface roughness, the cellular internalisation was elevated due to the advantage of a higher uptake of the rough surface nanoparticles loaded with drug for the treatment of melanoma [39].

#### 3.1.6. Hydrodynamic Size, Zeta Potential, and Polydispersity Index (PDI)

Particle size and zeta potential are the main parameters for nanoparticle performance, used to passively target therapeutic agents into cancer cells [40]. The tumour vasculature has a more heterogeneous distribution, higher vascular density, larger size, and is more permeable and leaky [41]. Many studies suggested that nanoparticles with a size smaller than 150 nm tend to extravasate from circulation and accumulate within tumours [42]. The experimental determination for the hydrodynamic diameter of the nCA was 120.1 ± 5.92 nm, and nCA/AFA was 105.6 ± 7.69 nm. The sizes classified both the nanocarrier and nanocomposite as promising candidates for passive targeting. Although, the hydrodynamic diameters for both nCA and nCA/AFA were higher than the HR-TEM sizes because the nanoparticles’ hydration layer is included within the calculated size [43,44], or possibly due to the aggregation of nanoparticles in deionised water [45].

**Figure 7:**
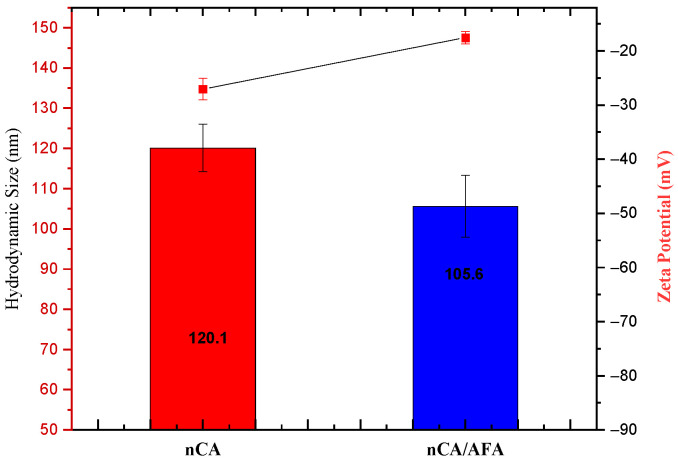
Average hydrodynamic diameter measurement (blue) and zeta potential (red).

The zeta potential was −27.03 ± 2.02 for nCA and −17.53 ± 1.12 mV for nCA/AFA (Figure 7). Changes in charge are considered an indirect confirmation of the conjugation of the nanocarrier with the drug. The negative charge surface for nano-formulation helps to prevent non-specific protein adsorption to the nanoparticle’s surface. The polydispersity indexes were 0.43 ± 0.03 and 0.29 ± 0.01 for nCA and nCA/AFA, respectively, indicating the good dispersal and homogeneity of the nanoparticles and nanocomposites [46].

#### 3.1.7. Surface Area and Porosity

**Figure 8:**
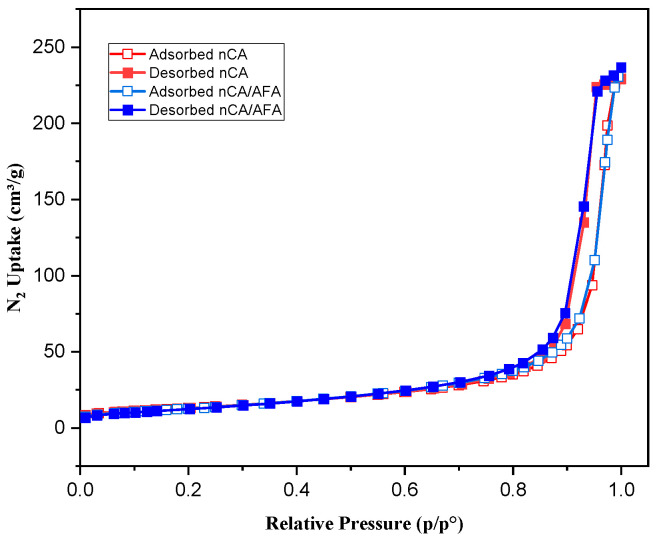
N_2_ isotherm for nCA (red) and nCA/AFA (blue). Open and solid squares represent adsorption and desorption, respectively.

N_2_ adsorption/desorption is a well-established analysis used to characterise porous solids and fine dry powders [47]. BET analysis measures the sample-specific surface area, including the pore size distribution, to estimate the dissolution rate and bioavailability [48]. Depending on the IUPAC classification system, the resulting isotherms from N_2_ adsorption/desorption obtained for nCA and nCA/AFA were type IV isotherms (Figure 8). The hysteresis loop was H1, indicating that the nanoparticles and nanocomposites are mesoporous [49]. The synthesised nCA- and nCA/AFA-specific surface areas, BIJ pore width, and pore volume are presented in Table 2. The decrease in surface area and pore volume for nCA/AFA confirms the drug’s loading into nanoparticles rather than on nanoparticle surfaces.

### 3.2. Yield, Encapsulation Efficiency, and Drug Loading

Table 3 presents information about the afatinib encapsulation efficiency (%), drug loading (%), and the yield (%) of different nanocomposites (nCA/AFA^1^–nCA/AFA^4^) according to the varying weight of afatinib used with 5 mg of nCA. The highest obtained DL% and EE% were 8.19% ± 0.52% and 55.08% ± 1.68% with 750 µg of afatinib. Furthermore, the EE% and DL% were noticed to be inversely reduced with increasing the afatinib weight up to 1000 µg, which can be explained as the maximum capacity of the nanocarrier at 750 µg. Such limitation suggests that the nanocomposite nCA/AFA^3^ can hold approximately 8.19 ± 0.52, and this was chosen as the maximum value for further characterisation and drug-release studies.

### 3.3. pH-Responsive Drug-Release Profile

#### 3.3.1. In Vitro Drug Release

**Figure 9:**
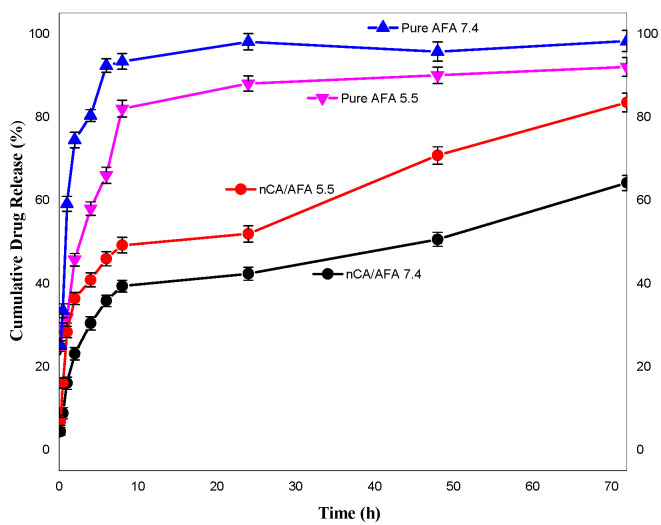
Release profile of pure AFA at pH 7.4 (blue), pure AFA at pH 5.5 (pink), nCA/AFA at pH 5.5 (red), and nCA/AFA at pH 7.4 (black).

For in vitro release, nCA/AFA and pure AFA samples were calculated at physiological (pH 7.4) and acidic (pH 5.5) conditions in PBS at 37 °C for 72 h. The release profile (Figure 9) demonstrates that after 1 h, 58.5% ± 2.5% and 32.04% ± 3.2% of pure AFA were released at pH 7.4 and 5.5, respectively. In contrast, AFA was released from nanocomposite nCA/AFA at pH 7.4, only 15.45% ± 3.9%, and 28.4% ± 3.1% at pH 5.5. Interestingly, AFA was released from nCA/AFA three-fold slower than the pure AFA [50]. Sequentially, at 72 h, 99.5% ± 8.3% and 83.3% ± 5.7% of pure AFA were released at pH 7.4 and 5.5 [22]. In comparison, after 72 h, 63.7% ± 5.1% and 81.3% ± 2.6% of AFA were released from nCA/AFA at pH 7.4 and 5.5, correspondingly. 

Pure AFA demonstrated a burst release profile at pH 7.4 and 5.5 at 1 h, while nCA/AFA displayed a sustained release profile, which was only 63.7% ± 5.1% up to 72 h. The drug-release data suggest that AFA is released from nCA in higher amounts and faster in an acidic environment tumour pH than physiological pH, which may be related to nCA pH-dependent solubility [34]. The carbonate apatite is dissociated at acidic pH, and the drug will accumulate at the tumour site. The sustained release of the drugs in a physiological pH environment is due to the nanoparticles’ physical structure and pores, which promote the release behaviour. This is similar to Hossain et al.’s release profile study, where the apatite nanoparticles were loaded with doxorubicin chemotherapy and had a sustained release profile [45]. The difference in the release is assumed to be due to the carbonate apatite’s hydrophilic nature with the afatinib, in comparison with the pure afatinib, which makes the molecules of liquid’s permeation into the nCA and the AFA to the external medium more complicated than pure AFA [51]. 

#### 3.3.2. Kinetics Drug Release

**Figure 10:**
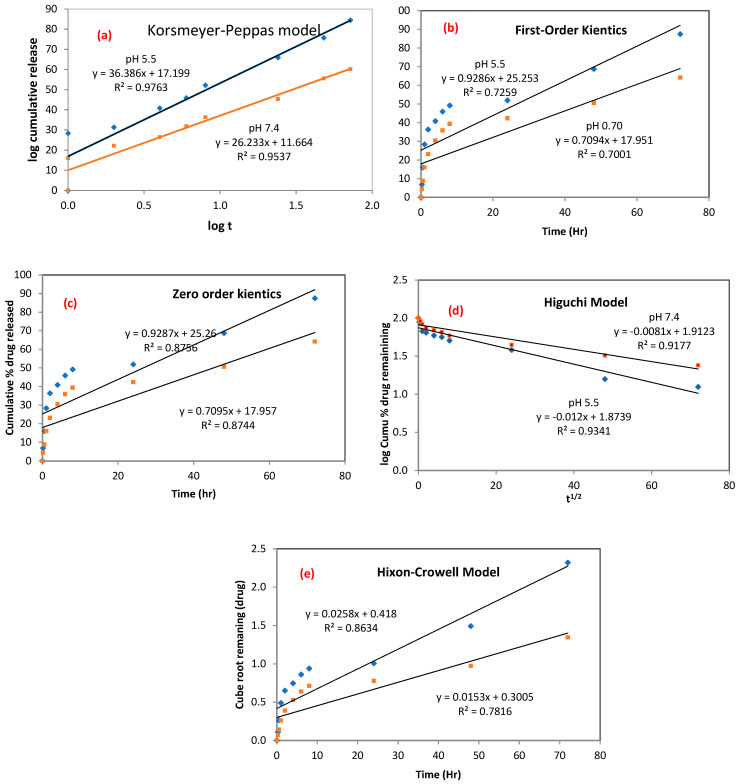
Kinetics drug release from nCA. (**a**) Korsmeyer–Peppas, (**b**) first-order kinetics, (**c**) zero-order kinetics, (**d**) Higuchi, and (**e**) Hixson–Crowell models.

The five pharmacokinetics models were applied to estimate the drug-release mechanism of nCA. As demonstrated in Figure 10a–e, five pharmacokinetics models were used to fit the experimental release, including Korsmeyer–Peppas, first-order kinetics, zero-order kinetics, Higuchi, and Hixson–Crowell. Varied fitting parameters, such as K_KP_, K_1_, K_0_, K_H_, and K_HC_, with the correlation coefficient for the kinetic model are shown in Table 4.

In the zero-order kinetics model, the amount of AFA drug released at a certain time will equal *q_e_,* and *q*_0_ is the initial amount of AFA used, while *t* and *K*_0_ represent the time and rection coefficients, respectively:

(5) $$q_{t}-q_{0}=K_{0}t$$

In the first-order kinetics model, *K*_1_ is the rate constant, as shown in Equation (6):

(6) $$\ln\;\left(q_{e}-q_{t}\right)=\ln q_{e}-K_{1}t$$

The Higuchi model studied the correlation between the log of AFA drug released versus the ascending square root of time, as shown in Equation (7):

(7) $$q_{t}=K_{H}\sqrt{t}$$

where *K_H_* is the rate constant of the Higuchi model.

Additionally, the Hixon–Crowell model demonstrates the relation between the cube root of AFA remaining in the nCA as a function of time, as shown in Equation (8):

(8) $$\sqrt[{3}]{\left(M_{0}-q_{t}\right)}=K_{HC}t$$

where *K_HC_* is the Hixon–Crowell rate constant and *M*_0_ is the initial drug concentration of AFA in nCA.

Finally, the Korsmeyer–Peppas model studied the relationship of the log of AFA released against the log of time:

(9) $$\log\;q_{t}=n\log t+\log K$$

where *q_t_* represents the fraction released by time, *t* (min), *n* represents the drug’s release exponent mechanism, and *K* (h-n) is the constant.

These mathematical and kinetics models demonstrated that afatinib release was proficient in nCA nanocarriers, and it was best fit to the Korsmeyer–Peppas model [47,48,52]. The Korsmeyer–Peppas model revealed the relationship between the AFA log released versus the time log and was adopted based on the release of the first 60% of the drug. This model has been previously used to describe the release kinetics of the gentamicin sulfate drug inside carbonate apatite nanocrystals [53]. The correlation coefficient values (R_2_) for the Korsmeyer–Peppas model were 0.9763 and 0.9537 for pH 5.5 and 7.4, respectively. The most significant mechanism involves a combination between diffusion and self-erosion of the matrix. The release exponent (*n*) can identify possible AFA release mechanisms. The determined *n* values were found to be 0.78 and 0.64 for pH 5.5 and 7.4, respectively. The *n* values were between 0.45 ≤ *n* ≤ 0.89, which indicates non-Fickian (anomalous) diffusion, as shown in Table 5 [54].

The highest *n* value was noticed at pH 5.5 (*n* = 0.78), demonstrating that the nCA surface was weakened over time by water influx, which facilitated ease of drug release and diffused from the nanocarrier into the dissolution media.

As per Mircioiu et al.’s study [55], the modelling and release kinetics predictions require a higher understanding of the physiological, physicochemical, and many mathematical aspects of the kinetics release. The in vitro release and dissolution studies were executed to determine the drug-release model for the synthesised nCA/AFA nanocomposite. It has been found that using a dialysis membrane to understand the release of the pure drug results in an underestimation because the drug is thought to dissolve in the dialysis compartment. This is why determining the release kinetics for pure AFA can be misleading compared to sustained release of AFA from nCA nanocarriers. The appearance of AFA released from nCA remained slow due to the slow permeation of AFA from the dialysis to the external solvent medium [56]. Generally, the dialysis flow rate of a drug is estimated via the solubility of the drug, the molecular size of the drug, and the pore size of the dialysis membrane. The flow rate will be faster if the drugs are hydrophilic, smaller, and the dialysis membrane has a large pore size [57]. In this study, the dialysis membrane used was 14,000 MWCO, which has an adequate pore size for the entry of solubilised afatinib (485.9) with a molar weight smaller than the pores of the dialysis membrane.

### 3.4. Dissolution Analysis of nCA in Biological Media

**Figure 11:**
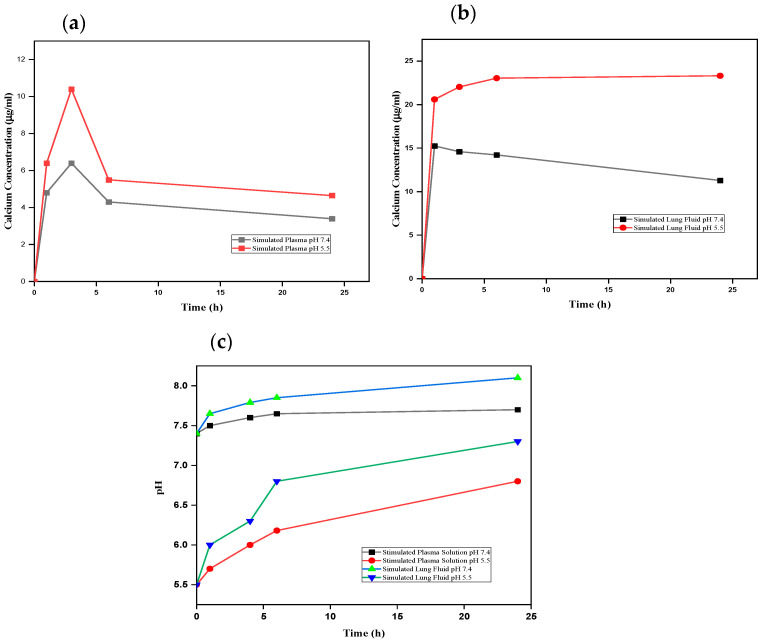
Dissolution analysis of nCA. (**a**) Calcium ion released from nCA in simulated plasma at pH 7.4 and 5.5. (**b**) Calcium ion released from nCA in simulated lung fluid at pH 7.4 and 5.5. (**c**) The alkalisation profile of nCA in both simulated plasma solution and simulated lung fluid at pH 7.4 and 5.5.

It is crucial to determine nCA’s interaction with different biological fluids to assess potential risks in the environmental medium. The nCA nanocarrier dissolution study was carried out in vitro using simulated plasma solution (SPS) and simulated lung fluid (SLF). The nCA was immersed at two different pH levels, 7.4 and 5.5, within different time intervals to study the concentration of calcium released. Figure 11a,b demonstrate higher dissolution profiles and higher calcium concentrations in acidic simulated plasma solution, similarly to simulated lung fluid. 

**Figure 12:**
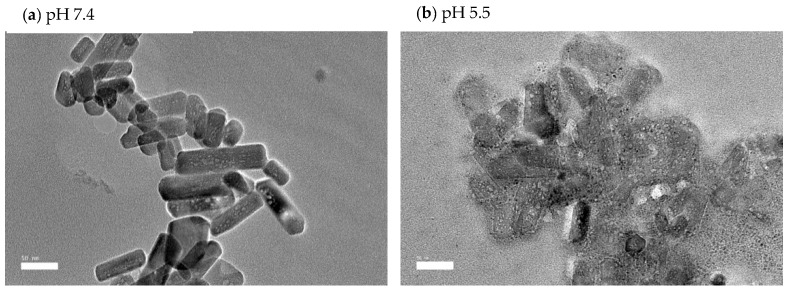
FESEM micrographs of nCA immersed in simulated lung fluid for 48 h. (**a**) nCA in simulated lung fluid at pH 7.4, (**b**) nCA in simulated lung fluid at pH 5.5.

The pH levels of all samples were determined to evaluate the alkalisation or neutralisation ability of the nCA, as shown in Figure 10c. The alkalisation abilities of the nCAs in both media were higher at pH 5.5 than at physiological pH 7.4. Ishikawa et al. [58] have proposed that carbonated hydroxyapatite nanoparticles increased the pH to about 7.4. The buffering capacity of nCA in both solutions at different pH levels confirms the nCA’s ability to alkalise the tumour microenvironment, which will help to decrease metastasis and tumour growth [58,59]. Since the function of calcium is the intracellular signal transducer, the extra calcium release from nCA may affect cellular signalling via increasing endoplasmic reticulum (ER) stress and induction of reactive oxygen species (ROS). However, calcium is the lowest toxic metal among others such as Ni^2+^, Cu^2+^, and Zn^2+^, even though it is an essential metal inside the human body and plays a critical role in regulating metabolism [59]. The FESEM micrographs of nCA in simulated lung fluid confirm the distortion of nanoparticles with reducing pH, as shown in Figure 12a,b. 

## 4. Conclusions

In conclusion, carbonate apatite nanoparticles were synthesised hydrothermally with appropriate morphology, size, porosity, and crystalline phase, and subsequently loaded with the targeted drug (afatinib) for treating non-small cell lung cancer. The resulting nanocomposite, termed nCA/AFA, was revealed to be successfully loaded with 8.19% ± 0.52% of the drug. Moreover, the drug release demonstrated sustained release within the highest solubilities in the acidic medium and alkalisation properties in simulated plasma and lung fluid. Furthermore, the drug release was kinetically fitted to the Korsmeyer–Peppas model, with an R^2^ of 0.97 and 0.95 for pH 5.5 and 7.4, respectively. Based on the overall results, the nCA is an excellent nanocarrier, and the nCa/AFA composite should have a place in cancer treatment and research.

## Figures and Tables

**Table 1:** AFM roughness analysis of nCA and nCA/AFA.

Sample	Ra (nm)	Rq (nm)	RMax (nm)	Skewness	Kurtosis	Surface Area Difference
nCA	0.582	0.809	7.8	1.90	3.89	1.7%
nCA/AFA	0.563	1.02	7.2	0.97	3.05	1.24%

**Table 2:** BET surface area, BJI pore size, and pore volume for nCA and nCA/AFA.

Sample Name	BET Specific Surface Area (m^2^/g)	BJH Mean Pore Width (nm)	Pore Volume (cm^3^/g)
nCA	55.53	7.88	0.11
nCA/AFA	52.61	7.36	0.09

**Table 3:** Encapsulation efficiency (%), drug loading (%), and yield of nanocomposites according to different afatinib weights used.

Sample	AFA Weight (µg)	EE (%)	DL (%)	Yield %
nCA/AFA^1^	150	15.27 ± 0.58	0.45 ± 0.47	45.38
nCA/AFA^2^	500	38.51 ± 0.83	3.84 ± 0.38	47.87
nCA/AFA^3^	750	55.08 ± 1.68	8.19 ± 0.52	50.81
nCA/AFA^4^	1000	41.44 ± 1.09	8.08 ± 0.05	49.86

**Table 4:** The kinetic parameters of AFA released from the nCA/AFA nanocomposite using different pharmacokinetics models.

Model	Korsmeyer–Peppas			First-Order Kinetics		Zero-Order Kinetics		Higuchi		Hixson–Crowell	
Name and pH of Medium	K_KP_	R^2^	n	K_1_	R^2^	K_0_	R^2^	K_H_	R^2^	K_HC_	R^2^
nCA/AFA pH 7.4	4.7461	0.9537	0.78	0.0705	0.7001	0.7001	0.8744	3.2501	0.9341	0.0304	0.8634
nCA/AFA pH 5.5	6.6638	0.9763	0.64	0.0742	0.7259	0.7258	0.8756	4.5278	0.9177	0.0291	0.7816

**Table 5:** The exponent of release (*n*) values and drug-release mechanisms.

Release Exponent (*n*)	Mechanism	Time
n≤0.45	Fickian diffusion	t^0.5^
0.45≤n≤0.89	Non-Fickian diffusion	t*^n−1^*
n≥0.89	Case II transport	t
n≥1	Super case II transport	t*^n−1^*

## Data Availability

Data can be provided upon request.

## References

[1] Sung H., Ferlay J., Siegel R.L., Laversanne M., Soerjomataram I., Jemal A., Bray F. (2021). Global Cancer Statistics 2020: GLOBOCAN Estimates of Incidence and Mortality Worldwide for 36 Cancers in 185 Countries. CA A Cancer J. Clin..

[2] Newman S.P. (2017). Drug Delivery to the Lungs: Challenges and Opportunities. Ther. Deliv..

[3] Zappa C., Mousa S.A. (2016). Non-Small Cell Lung Cancer: Current Treatment and Future Advances. Transl. Lung Cancer Res..

[4] Kutkowska J., Porębska I., Rapak A. (2017). Non-Small Cell Lung Cancer—Mutations, Targeted and Combination Therapy. Postepy Hig. Med. Dosw. (Online).

[5] Zhu T., Bao X., Chen M., Lin R., Zhuyan J., Zhen T., Xing K., Zhou W., Zhu S. (2020). Mechanisms and Future of Non-Small Cell Lung Cancer Metastasis. Front. Oncol..

[6] Senapati S., Mahanta A.K., Kumar S., Maiti P., Kumar Mahanta A., Kumar S., Maiti P. (2018). Controlled Drug Delivery Vehicles for Cancer Treatment and Their Performance.

[7] Senapati S., Mahanta A.K., Kumar S., Maiti P. (2018). Controlled Drug Delivery Vehicles for Cancer Treatment and Their Performance. Signal Transduct. Target. Ther..

[8] Soares S., Sousa J., Pais A., Vitorino C. (2018). Nanomedicine: Principles, Properties, and Regulatory Issues. Front. Chem..

[9] Zhang Y., Li M., Gao X., Chen Y., Liu T. (2019). Nanotechnology in Cancer Diagnosis: Progress, Challenges and Opportunities. J. Hematol. Oncol..

[10] Wu X., Yamamoto H., Nakanishi H., Yamamoto Y., Inoue A., Tei M., Hirose H., Uemura M., Nishimura J., Hata T. (2015). Innovative Delivery of SiRNA to Solid Tumors by Super Carbonate Apatite. PLoS ONE.

[11] Hossain S.M., Shetty J., Tha K.K., Chowdhury E.H. (2019). α-Ketoglutaric Acid-Modified Carbonate Apatite Enhances Cellular Uptake and Cytotoxicity of a Raf- Kinase Inhibitor in Breast Cancer Cells through Inhibition of MAPK and PI-3 Kinase Pathways. Biomedicines.

[12] Islam R.A., Al-Busaidi H., Zaman R., Abidin S.A.Z., Othman I., Chowdhury E.H. (2020). Carbonate Apatite and Hydroxyapatite Formulated with Minimal Ingredients to Deliver SiRNA into Breast Cancer Cells in Vitro and in Vivo. J. Funct. Biomater..

[13] Vermorken J.B., Rottey S., Ehrnrooth E., Pelling K., Lahogue A., Wind S., Machiels J.P. (2013). A Phase Ib, Open-Label Study to Assess the Safety of Continuous Oral Treatment with Afatinib in Combination with Two Chemotherapy Regimens: Cisplatin plus Paclitaxel and Cisplatin plus 5-Fluorouracil, in Patients with Advanced Solid Tumors. Ann. Oncol..

[14] Maarof N.N.N., Alsalahi A., Abdulmalek E., Fakurazi S., Tejo B.A., Rahman M.B.A. (2021). Efficacy of Afatinib in the Treatment of Patients with Non-Small Cell Lung Cancer and Head and Neck Squamous Cell Carcinoma: A Systematic Review and Meta-Analysis. Cancers.

[15] Yoshioka H., Kato T., Okamoto I., Tanaka H., Hida T., Seto T., Kiura K., Tian Y., Azuma H., Yamamoto N. (2020). Therapies after First-Line Afatinib in Patients with EGFRm(+) NSCLC in Japan: Retrospective Analysis of LUX-Lung 3. Future Oncol..

[16] Reguart N., Remon J. (2015). Common EGFR-Mutated Subgroups (Del19/L858R) in Advanced Non-Small-Cell Lung Cancer: Chasing Better Outcomes with Tyrosine Kinase Inhibitors. Future Oncol. (Lond. Engl.).

[17] Engelman J.A., Jänne P.A. (2008). Mechanisms of Acquired Resistance to Epidermal Growth Factor Receptor Tyrosine Kinase Inhibitors in Non-Small Cell Lung Cancer. Clin. Cancer Res. Off. J. Am. Assoc. Cancer Res..

[18] Cryer A.M., Chan C., Eftychidou A., Maksoudian C., Mahesh M., Tetley T.D., Spivey A.C., Thorley A.J. (2019). Tyrosine Kinase Inhibitor Gold Nanoconjugates for the Treatment of Non-Small Cell Lung Cancer. ACS Appl. Mater. Interfaces.

[19] Hirsh V. (2015). Next-Generation Covalent Irreversible Kinase Inhibitors in NSCLC: Focus on Afatinib. BioDrugs Clin. Immunother. Biopharm. Gene Ther..

[20] Yang J.C., Wu Y.L., Schuler M., Sebastian M., Popat S., Yamamoto N., Zhou C., Hu C.P., O’Byrne K., Feng J. (2015). Afatinib versus Cisplatin-Based Chemotherapy for EGFR Mutation-Positive Lung Adenocarcinoma (LUX-Lung 3 and LUX-Lung 6): Analysis of Overall Survival Data from Two Randomised, Phase 3 Trials. Lancet Oncol..

[21] Ichiki M., Wataya I., Yamada K., Tsuruta N., Takeoka H., Okayama Y., Sasaki J., Hoshino T. (2017). Preventive Effect of Kampo Medicine (Hangeshashin-to, TJ-14) plus Minocycline against Afatinib-Induced Diarrhea and Skin Rash in Patients with Non-Small Cell Lung Cancer. Oncotargets Ther..

[22] Elbatanony R.S., Parvathaneni V., Kulkarni N.S., Shukla S.K., Chauhan G., Kunda N.K., Gupta V. (2021). Afatinib-Loaded Inhalable PLGA Nanoparticles for Localized Therapy of Non-Small Cell Lung Cancer (NSCLC)-Development and in-Vitro Efficacy. Drug Deliv. Transl. Res..

[23] Hong S.T., Lin H., Wang C.S., Chang C.H., Lin A.M.Y., Yang J.C.H., Lo Y.L. (2019). Improving the Anticancer Effect of Afatinib and MicroRNA by Using Lipid Polymeric Nanoparticles Conjugated with Dual PH-Responsive and Targeting Peptides. J. Nanobiotechnol..

[24] Mourdikoudis S., Pallares R.M., Thanh N.T. (2018). Characterization Techniques for Nanoparticles: Comparison and Complementarity upon Studying Nanoparticle Properties. Nanoscale.

[25] Ren F., Leng Y., Ding Y., Wang K. (2013). Hydrothermal Growth of Biomimetic Carbonated Apatite Nanoparticles with Tunable Size, Morphology and Ultrastructure. CrystEngComm.

[26] Rasuli H., Rasuli R., Alizadeh M., BoonTong G. (2020). Microwave-Assisted Exfoliation and Tearing of Graphene Oxide in the Presence of TiO_2_ Nanoparticles. Results Phys..

[27] Lu X.Y., Liu S., Han M.S., Yang X.C., Sun K.X., Wang H.B., Mu H.J., Du Y., Wang A.P., Ni L. (2019). Afatinib-Loaded Immunoliposomes Functionalized with Cetuximab: A Novel Strategy Targeting the Epidermal Growth Factor Receptor for Treatment of Non-Small-Cell Lung Cancer. Int. J. Pharm..

[28] Chemmalar S., Intan-Shameha A.R., Abdullah C.A.C., Razak N.A.A., Yusof L.M., Ajat M., Gowthaman N.S.K., Bakar M.Z.A. (2021). Synthesis and Characterization of Gefitinib and Paclitaxel Mono and Dual Drug-Loaded Blood Cockle Shells (Anadara Granosa)-Derived Aragonite CaCO_3_ Nanoparticles. Nanomaterials.

[29] Lara-Ochoa S., Ortega-Lara W., Enrique Guerrero-Beltrán C., Pinto Ribeiro V., Miguel Oliveira J., Reis R.L. (2021). Hydroxyapatite Nanoparticles in Drug Delivery: Physicochemistry and Applications. Pharmaceutics.

[30] Martínez-Casado F.J., Iafisco M., Delgado-López J.M., Martínez-Benito C., Ruiz-Pérez C., Colangelo D., Oltolina F., Prat M., Gómez-Morales J. (2016). Bioinspired Citrate-Apatite Nanocrystals Doped with Divalent Transition Metal Ions. Cryst. Growth Des..

[31] Gómez-Morales J., Verdugo-Escamilla C., Fernández-Penas R., Parra-Milla C.M., Drouet C., Maube-Bosc F., Oltolina F., Prat M., Fernández-Sánchez J.F. (2018). Luminescent Biomimetic Citrate-Coated Europium-Doped Carbonated Apatite Nanoparticles for Use in Bioimaging: Physico-Chemistry and Cytocompatibility. RSC Adv..

[32] Sari M., Kristianto N.A., Chotimah, Ana I.D., Yusuf Y. (2021). Carbonated Hydroxyapatite-Based Honeycomb Scaffold Coatings on a Titanium Alloy for Bone Implant Application—Physicochemical and Mechanical Properties Analysis. Coatings.

[33] Mozar F.S., Chowdhury E.H. (2017). Surface-Modification of Carbonate Apatite Nanoparticles Enhances Delivery and Cytotoxicity of Gemcitabine and Anastrozole in Breast Cancer Cells. Pharmaceutics.

[34] Mozar F., Chowdhury E. (2015). Gemcitabine Interacts with Carbonate Apatite with Concomitant Reduction in Particle Diameter and Enhancement of Cytotoxicity in Breast Cancer Cells. Curr. Drug Deliv..

[35] Syazwan Mohd Noor M., Nabilah Afja Mohd Afandi N., Mohd Noor A.-F., Marliana Baba Ismail Y., Marliana Baba Y. (2020). Effect of Carbonate to Phosphate Molar Ratios on the Physico-Chemical Properties of Carbonated Hydroxyapatite Nanopowder. J. Eng. Sci..

[36] Webb H.K., Truong V.K., Hasan J., Fluke C., Crawford R.J., Ivanova E.P. (2012). Roughness Parameters for Standard Description of Surface Nanoarchitecture. Scanning.

[37] Hashemzadeh H., Allahverdi A., Sedghi M., Vaezi Z., Moghadam T.T., Rothbauer M., Fischer M.B., Ertl P., Naderi-Manesh H. (2020). PDMS Nano-Modified Scaffolds for Improvement of Stem Cells Proliferation and Differentiation in Microfluidic Platform. Nanomaterials.

[38] Xue J., Zhu Y., Bai S., He C., Du G., Zhang Y., Zhong Y., Chen W., Wang H., Sun X. (2021). Nanoparticles with Rough Surface Improve the Therapeutic Effect of Photothermal Immunotherapy against Melanoma. Acta Pharm. Sin. B.

[39] Aw-Yong P.Y., Gan P.H., Sasmita A.O., Mak S.T., Ling A. (PDF) Nanoparticles as Carriers of Phytochemicals: Recent Applications against Lung Cancer | Andrew O Sasmita—Academia.Edu. https://www.academia.edu/35949766/Nanoparticles_as_Carriers_of_Phytochemicals_Recent_Applications_Against_Lung_Cancer.

[40] Maeda H. (2015). Toward a Full Understanding of the EPR Effect in Primary and Metastatic Tumors as Well as Issues Related to Its Heterogeneity. Adv. Drug Deliv. Rev..

[41] Eaton P., Quaresma P., Soares C., Neves C., De Almeida M.P., Pereira E., West P. (2017). A Direct Comparison of Experimental Methods to Measure Dimensions of Synthetic Nanoparticles. Ultramicroscopy.

[42] Kamal N.A.M.A., Abdulmalek E., Fakurazi S., Cordova K.E., Abdul Rahman M.B. (2021). Surface Peptide Functionalization of Zeolitic Imidazolate Framework-8 for Autonomous Homing and Enhanced Delivery of Chemotherapeutic Agent to Lung Tumor Cells. Dalton Trans..

[43] Souza T.G.F., Ciminelli V.S.T., Mohallem N.D.S. (2016). A Comparison of TEM and DLS Methods to Characterize Size Distribution of Ceramic Nanoparticles.

[44] Danaei M., Dehghankhold M., Ataei S., Hasanzadeh Davarani F., Javanmard R., Dokhani A., Khorasani S., Mozafari M.R. (2018). Impact of Particle Size and Polydispersity Index on the Clinical Applications of Lipidic Nanocarrier Systems. Pharmaceutics.

[45] Tan Y.H., Davis J.A., Fujikawa K., Ganesh N.V., Demchenko A.V., Stine K.J. (2012). Surface Area and Pore Size Characteristics of Nanoporous Gold Subjected to Thermal, Mechanical, or Surface Modification Studied Using Gas Adsorption Isotherms, Cyclic Voltammetry, Thermogravimetric Analysis, and Scanning Electron Microscopy. J. Mater. Chem..

[46] Abbasi Aval N., Pirayesh Islamian J., Hatamian M., Arabfirouzjaei M., Javadpour J., Rashidi M.R. (2016). Doxorubicin Loaded Large-Pore Mesoporous Hydroxyapatite Coated Superparamagnetic Fe3O4 Nanoparticles for Cancer Treatment. Int. J. Pharm..

[47] Cychosz K.A., Thommes M. (2018). Progress in the Physisorption Characterization of Nanoporous Gas Storage Materials. Engineering.

[48] Lo Y.L., Lin H.C., Hong S.T., Chang C.H., Wang C.S., Lin A.M.Y. (2021). Lipid Polymeric Nanoparticles Modified with Tight Junction-Modulating Peptides Promote Afatinib Delivery across a Blood–Brain Barrier Model. Cancer Nanotechnol..

[49] Fosca M., Rau J.V., Uskoković V. (2022). Factors Influencing the Drug Release from Calcium Phosphate Cements. Bioact. Mater..

[50] Sambudi N.S., Cho S., Cho K. (2016). Porous Hollow Hydroxyapatite Microspheres Synthesized by Spray Pyrolysis Using a Microalga Template: Preparation, Drug Delivery, and Bioactivity. RSC Adv..

[51] Hesaraki S., Zamanian M., Khorami M. Nano-Structured Apatite Granules as Drug Delivery Device for Bone Infection Treatments. https://www.researchgate.net/publication/229038005_Nano-Structured_Apatite_Granules_as_Drug_Delivery_Device_for_Bone_Infection_Treatments.

[52] Unagolla J.M., Jayasuriya A.C. (2018). Drug Transport Mechanisms and In Vitro Release Kinetics of Vancomycin Encapsulated Chitosan-Alginate Polyelectrolyte Microparticles as a Controlled Drug Delivery System. Eur. J. Pharm. Sci. Off. J. Eur. Fed. Pharm. Sci..

[53] Mircioiu C., Voicu V., Anuta V., Tudose A., Celia C., Paolino D., Fresta M., Sandulovici R., Mircioiu I. (2019). Mathematical Modeling of Release Kinetics from Supramolecular Drug Delivery Systems. Pharmaceutics.

[54] Yu M., Yuan W., Li D., Schwendeman A., Schwendeman S.P. (2019). Predicting Drug Release Kinetics from Nanocarriers inside Dialysis Bags. J. Control. Release Off. J. Control. Release Soc..

[55] Ma-Ham A., Wu H., Wang J., Kang X., Zhang Y., Lin Y. (2011). Apoferritin-Based Nanomedicine Platform for Drug Delivery: Equilibrium Binding Study of Daunomycin with DNA. J. Mater. Chem..

[56] Ishikawa K., Hayashi K. (2021). Carbonate Apatite Artificial Bone. Sci. Technol. Adv. Mater..

[57] Som A., Raliya R., Tian L., Akers W., Ippolito J.E., Singamaneni S., Biswas P., Achilefu S. (2016). Monodispersed Calcium Carbonate Nanoparticles Modulate Local PH and Inhibit Tumor Growth in Vivo. Nanoscale.

[58] Cieplik F., Rupp C.M., Hirsch S., Muehler D., Enax J., Meyer F., Hiller K.A., Buchalla W. (2020). Ca^2+^ Release and Buffering Effects of Synthetic Hydroxyapatite Following Bacterial Acid Challenge. BMC Oral Health.

[59] Horie M., Nishio K., Kato H., Endoh S., Fujita K., Nakamura A., Kinugasa S., Hagihara Y., Yoshida Y., Iwahashi H. (2014). Evaluation of Cellular Influences Caused by Calcium Carbonate Nanoparticles. Chem.-Biol. Interact..

