# Correction: Maarof et al. Biodegradable Carbonate Apatite Nanoparticle as a Delivery System to Promote Afatinib Delivery for Non-Small Cell Lung Cancer Treatment. *Pharmaceutics* 2022, *14*, 1230

**DOI:** 10.3390/pharmaceutics16101279

**Published:** 2024-09-30

**Authors:** Nian N. N. Maarof, Emilia Abdulmalek, Sharida Fakurazi, Mohd Basyaruddin Abdul Rahman

**Affiliations:** 1 Integrated Chemical BioPhysics Research, Department of Chemistry, Faculty of Science, Universiti Putra Malaysia, Serdang 43400, Selangor, Malaysia; gs53758@student.upm.edu.my (N.N.N.M.); emilia@upm.edu.my (E.A.); 2 Department of Chemistry, College of Education, University of Sulaimani, Sulaimani 46001, Iraq; 3 Laboratory of Vaccines and Immunotherapeutics, Institute of Bioscience, Universiti Putra Malaysia UPM, Serdang 43400, Selangor, Malaysia; sharida@upm.edu.my; 4 Department of Human Anatomy, Faculty of Medicine and Health Sciences, Universiti Putra Malaysia UPM, Serdang 43400, Selangor, Malaysia; 5 UPM-MAKNA Cancer Laboratory, Institute of Bioscience, Universiti Putra Malaysia, Serdang 43400, Selangor, Malaysia

In the original publication, there was a mistake in the legend for **“Figure 3”**. The transmittance changes due to afatinib loading into nCA are mentioned in the green box. The correct legend appears below. The authors state that the scientific conclusions are unaffected. This correction was approved by the Academic Editor. The original publication has also been updated.

**Figure 3**. FTIR spectra for nCA (red), nCA/AFA (blue), and AFA (green).
